# Hydrogen enhanced biodiesel production from Botryococcus braunii algal oil for sustainable fuel development

**DOI:** 10.1038/s41598-026-40516-4

**Published:** 2026-02-18

**Authors:** M. Selvam, Pragadish Nagarajan, K. A. Harish, V. Dhivya, R. Ganapathy Srinivasan, S. P. Samal, Ashish Agrawal

**Affiliations:** 1 Department of Mechanical Engineering, Vel Tech Multi Tech Dr.Rangarajan Dr.Sakunthala Engineering College, Avadi, 600062 India; 2 Department of Mechanical Engineering, Rajalakshmi Institute of Technology, Chennai, 600124 India; 3 Department of Electronics and Communication Engineering, Vel Tech Multi Tech Dr.Rangarajan Dr.Sakunthala Engineering College, 600062 Avadi, India; 4 Department of Biosciences, Saveetha School of Engineering. Saveetha Institute of Medical and Technical Sciences, Chennai, 602105 India; 5 Manipal Institute of Technology, Manipal Academy of Higher Education, Manipal, India

**Keywords:** Botryococcus braunii biodiesel, Sustainable diesel engine fuel, Hydrogen blending, Brake thermal efficiency (BTE), Combustion efficiency, Specific fuel consumption (SFC), Carbon monoxide emissions, Energy science and technology, Engineering, Environmental sciences

## Abstract

The growing demand have made researchers look into algae-based biodiesel, which is precisely a type of algae called Botryococcus braunii, as an alternate to sustainable fuel source. The feature of this alga is that it has high oil content and is determined as user friendly. The study made an attempt to explore the effectiveness and efficiency of adding hydrogen with Botryococcus braunii at 2 different rates, viz., 4LPM and 8LPM respectively in a Common Rail Direct Injection (CRDI) diesel engine and compared its performance with the pure diesel. This assessment approach of testing hydrogen (H_2_) with 30% Botryococcus braunii biodiesel + 70% diesel blend combination (A30) and comparing the same with pure diesel is regarded as the primary creativity and uniqueness of this research investigation. The analysis of the study revealed the fact that adding hydrogen to 30% Botryococcus braunii biodiesel + 70% diesel blend enhanced the performance of the engine substantially. It further demonstrated that the Brake Thermal Efficiency improved to 37% from 31% with the addition of A30 + H_2_ (8 LPM) against the pure diesel, marking a rise of 19.35%. Similarly, specific fuel consumption dropped to 0.24 kg/kWh from 0.30 kg/kWh, illustrating a reduction of 20%, in comparison with pure diesel. Volumetric efficiency surged to 91% (with a rise of 10.98%) from 82%. This is because of the presence and availability of more content of oxygen in 30% Botryococcus braunii biodiesel + 70% diesel blend, facilitating a better and a clean process of combustion. The study focused on the properties of combustion improved with the in-cylinder pressure at the utmost load, where it was found rising to 77.3 bar from 70.2 bar, indicating a marginal surge of 10.11% against the net heat release rate, demonstrating a steep rise of 18.26% (increased to 48.63 kJ/m^3^deg from 41.11 kJ/m³deg). Besides the above, the Carbon Monoxide (CO) emissions reduced by 69.46% (0.334% to 0.102%), Hydrocarbons decreased by 43.24% (148ppm to 84ppm), CO_2_ dropped by 7.77% (10.3% to 9.5%), smoke opacity diminished by 14.18% (69.1% to 59.3). It was evidently observed that the hydrogen blend algae biodiesel enhanced a better combustion process by the removal of incomplete combustion areas and enhancing the overall performance and efficiency of the engine. It is noted that while the outcomes of the emissions reveal substantial enhancements, Nitrogen oxides (NO_X_) emissions do increase by 47.64% (from 1503ppm to 2220ppm). The exhaust gas temperature is decreased marginally from 520 °C to 498 °C, marking a reduction of 4.23%. In a nutshell, the study apparently reveals the fact that A30 + H_2_ (8 LPM) significantly improves the performance of the engine, enhances the characteristics of combustion and minimizes most emissions leading to a very high and an efficient sustainable fuel in contrast to the pure diesel.

## Introduction

 Multiple research studies which analyze the biodiesel blends derived from different sources to develop and substitute the diesel fuels for automotive applications. When compared to traditional diesel fuel^1^. Studies have shown the waste cooking oil in combination with cedar wood oil. It also produces the beneficial combustion results along with a decreased emissions. Research indicates that an optimized blends using palm-oil biodiesel with diethyl ether and ethanol lead to NO_X_, CO and hydrocarbons (HC) decreases together without altering any kind of diesel-like noise levels^2^. Hydrogen-enriched biodiesel–ethanol blends in a CI engine were shown to improve brake thermal efficiency, enhance combustion characteristics, and significantly reduce carbon monoxide and unburned hydrocarbon emissions^3^. The performance characteristics of Botryococcus braunii algal biodiesel have obtained through solvent extraction. It matches the diesel fuel based on research findings^4^. Studies on hydrogen-assisted ethanol–biodiesel operation demonstrated improved engine performance, reduced exhaust emissions, and enhanced exergy efficiency under optimized hydrogen induction conditions^5^. Hydrogen addition in diesel–biodiesel–ethanol engines was reported to promote effective NOx reduction through selective catalytic reduction, highlighting hydrogen’s influence on emission mitigation mechanisms^6^. Successful implementation of CRDI engine technology occurs while using the biodiesel made from waste cooking oil which delivers the better operational performance while diminishing exhaust emissions^7^Experimental research by RSM demonstrates that algal biodiesel percentages in blends creates a better CO and HC emissions which controls and also increases NO_X_ and carbon dioxide (CO_2_) emissions^8^. When Scenedesmus obliquus biodiesel is blended with n-Butanol the overall brake thermal efficiency rises while both CO and HC emissions decreases at the cost of increased NOx production^9^. Experimental investigations on hydrogenated biodiesel–ethanol–diesel ternary blends revealed improved combustion behavior, reduced regulated emissions, and noticeable variations in unregulated exhaust pollutants^10^. The combined addition of hydrogen and ethanol to biodiesel-fueled DI diesel engines resulted in enhanced combustion efficiency, improved thermal performance, and reduced carbon-based exhaust emissions^11^. The performance of microalgae biodiesel blend A30 matches the diesel power levels yet to produce the considerable emissions reductions^12^. According to scientific research^13^B40 biodiesel blends to show the performance levels highly to increase when it has been used to be coated piston, when compared to conventional piston engine that based on most tested parameters. The timing of fuel injection has shown promise in maximizing the combustion efficiency of algal biodiesel blends according to the research^14^but the analysis of emissions shows NO_X_ emissions will be continued to be a major concern^15^. Studies of biodiesel blends at their best ratio that shown the performance staying within an acceptable ranges and offered significant emission reductions of CO and HC^16^. Research shows that biodiesel derived from Scenedesmus obliquus through transesterification yields better operational efficiency after addition in n-pentane^17^. The emissions reductions from algae cultivation and oil extraction and biodiesel transesterification processes lack an evidence on long-term engine durability^18^. According to using algae biodiesel^19^. Experimental analysis of algae biodiesel mixtures have indicated they also improved the combustion performance while decreasing the carbon monoxide and hydrocarbon emissions. Research shows A30 stands out at the most efficient option between the performance and emissions which results in paraffinic blend usage^20^. Biofuels for compression ignition engines have demonstrated a direct relationship between rising biodiesel content and decreased smoke opacity according to a complete engine analysis^21^. Algae-derived biodiesel blends have produced to improve the fuel efficiency while releasing fewer emissions when nanotechnology additives have increased^22^. A proper adjustment of blend ratios alongside engine load management and injection timing schedules leads to enhance the performance together with reduced emissions^23^. The carbon emissions from microalgae-based biodiesel production remains nearly to the nonexistent while researchers aim to optimize the system efficiency while minimizing NO_X_ emissions^24^. Because the direct injection engines uses Botryococcus braunii biodiesel their metrics have aligned closely with diesel performance yet to produce higher NO_X_ emissions^25^. Hydrogen-enriched biodiesel-based dual-fuel operation showed notable improvements in brake thermal efficiency and reductions in CO and HC emissions, though an increase in NOx emissions was observed^26^. Research on mixotrophic microalgae-derived biodiesel emphasized high lipid productivity, sustainability advantages, and strong potential as an alternative clean fuel for future diesel engine applications^27^. Laboratory tests revealed that increasing algae biodiesel concentrations have results in improved soot field and transparency along with decreased particulate emissions but led to moderate temperature rises^28^. The environmental advantages of algae-derived biofuels have received extensive documentation and blend ratios which serves to maintain the performance and minimize the emissions^29^. The use of algae-based biodiesel represents a strong alternative to conventional fossil fuels in modern applications. Combustion efficiency improves in engine performance while emissions like NO_X_ remain slightly elevated but other emissions shows a decreased amounts. Hydrogen with Botryococcus braunii algae oil biodiesel has proved to enhance brake thermal efficiency and decrease fuel consumption. Thus establishing an environmentally friendly diesel engine alternative.

To find sustainable and environmentally friendly alternatives to non-fossil and environmentally friendly fuels has generated a major interest in microalgae-derived biodiesel, due to its renewable natural and combustible properties. Recent research have revealed the potential for an increase in hydrogen in biodiesel blends to increase engine performance and control harmful emissions. Kumar^30^published a review paper on Biodiesel Additives have attained from various refrigerants with addition of Hydrogen. It also presented an improvements in combustion efficiency, brake thermal efficiency and reduced CO and HC emissions. Lawrence et al.^31^investigated that hydrogen lead addition to nano-enhanced lemongrass biodiesel blends and reports the thermal efficiency improvement and limited pollution of generation in compression ignition engines. Further, Jeyapaul and Shivanraju^32^investigated biodiesel, bioethanol and nanoparticle mixtures with hydrogen addition and showes the combination of nanoparticles and hydrogen which reduced the engine emissions and preserved performance. Ahmad et al.^33^using Al_2_O_3_ nanoparticles and hydrogen as a secondary fuel in Scenedesmus dimorphus microalgae biodiesel, found the higher cylinder pressure, improves a heat release rate and lower carbon monoxide emissions. Devanesan et al.^34^showed that TiO_2_ nanoparticles and hydraulic biodiesel mixtures with hydrogen-rich biogas improved an irritant stability, reduced particulate emissions and improved brake thermal efficiency. All of these studies shows that combination of microalgae biodiesel with hydrogen and nanoparticles can improve the fuel ignition and create a new way for sustainable, low-pollution output. This review conducted with the aim of testing the biodiesel compounds of the superiority of Botryococcus braunii, emphasizing its high lipid content, hydrocarbon-rich structure, and suitability for sustainable biodiesel production and hydrogen enhancement of Botryochus braunii algae oil, improving an engine efficiency and emission reduction, contributes to the development of the next generation of green fuels.

### Research gap and objectives

Existing in the research coverage of biodiesel from different feedstocks remains lacking when it comes to analyzing the performance and emission data from Botryococcus braunii algae oil. Research into algal biodiesel made from Botryococcus braunii needs a further investigation aside from existing exploration of hydrogen blending to enhance the diesel engine performance and reduce emissions. Current research discusses the problems with algal biodiesel emissions and lower calorific value yet to propose the minimal solutions which optimizes the performance and emission metrics. The study investigates how the engine performance and emission output changes when hydrogen is added to 30% *Botryococcus braunii* biodiesel + 70% diesel (A30 + H_2_). The research compares Pure Diesel Fuel (D100) with A30 biodiesel and hydrogen that blend the fuels under 4 LPM and 8 LPM flow conditions to evaluate their brake thermal efficiency and fuel consumption. It also examine the emission patterns for CO and CO_2_ along with determining how the hydrogen addition enhances in engine performance and supports in environmental friendly diesel operation.

### Resources and techniques

#### Production of biodiesel

**Fig. 1:**
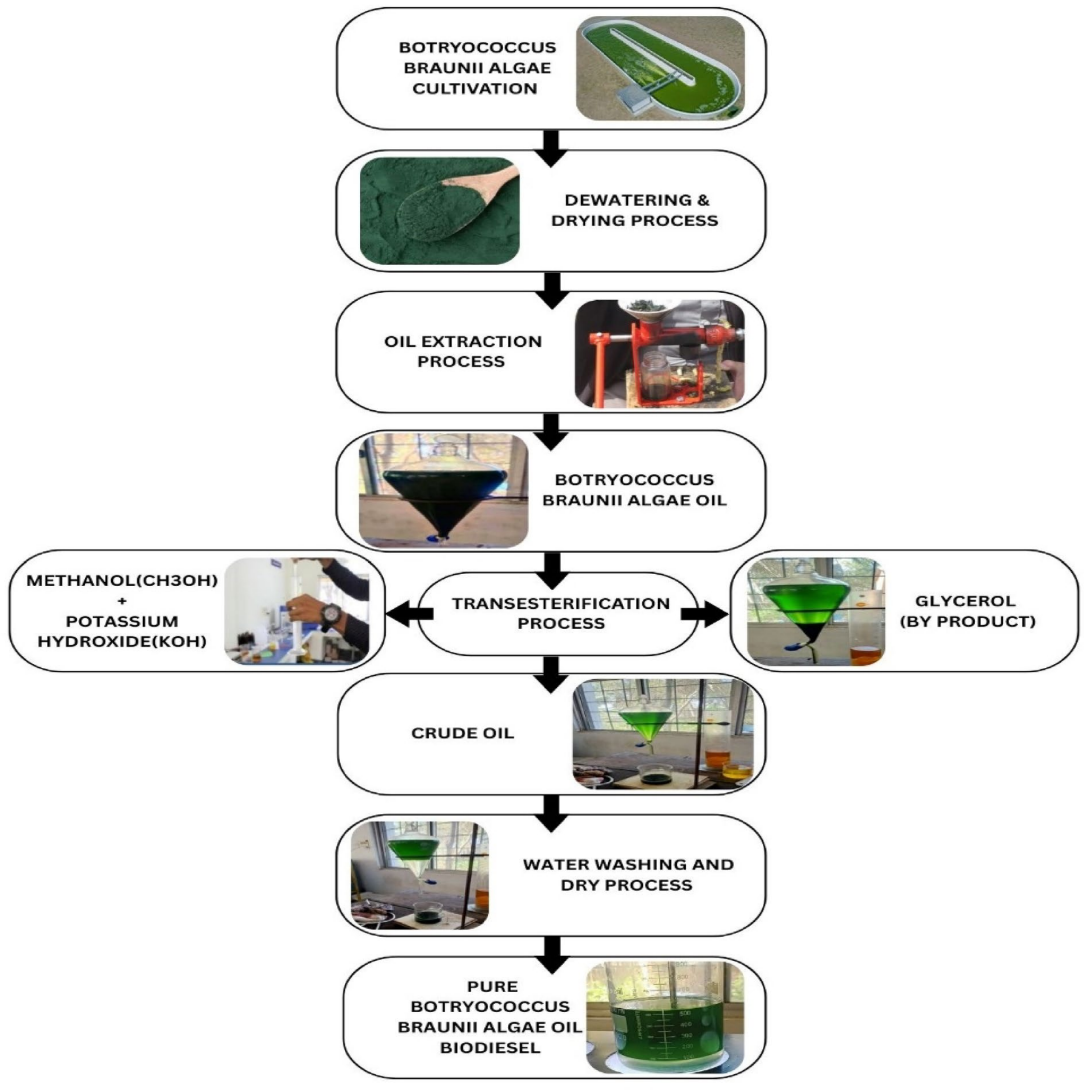
Production flowchart.

The production of biodiesel from Botryococcus braunii algae occurred through controlled procedures. The introduction of carbon dioxide into growth medium as a photosynthetic stimulant increased both photosynthetic output and biomass production. The cultivation spanned from 15 to 18 days until the cells reached maximum lipid levels where harvesting began with a flotation filtration process plus centrifugation method. The biomass dewatering procedure through this method effectively saved both power usage and material waste. The process of drying follows harvesting since it serves as a vital step towards lipid extraction. Double drying by solar power and ovens reduced the biomass moisture level to under 10% before grinding the dried material which optimized the extraction yield. Multiple factors were considered for choosing the hexane and ethanol (2:1) mixture to extract lipids since it effectively dissolves lipids for separation purposes Soxhlet extraction occurred for 6–8 h to reach a 90% moderate oil yield efficiency before distilling the crude algae oil which yielded a greenish-yellow viscous oil. The crude oil received preliminary purification for removing both free fatty acids (FFAS) and residual moisture from its composition. The crude oil obtained refined using techniques of degumming and subsequent neutralization with calcium oxide before vacuum drying. A biodiesel production method was implemented after the purification of the oil to create biodiesel from the refined substance. A reaction between Botryococcus braunii algae oil and methanol using Potassium hydroxide (KOH) catalysis enabled the conversion of triglycerides to fatty acid methyl esters (FAMES) which make up most of biodiesel production occurred through 2 h at 80 °C with continuous stirring. The biodiesel was repeatedly washed with warm distilled water until the wash water became clear, effectively removing residual methanol, catalysts, and impurities. It was then dried under a vacuum to give clear golden-yellow fuel with properties similar to those of conventional pure diesel. Any remaining trace water was removed by heating the biodiesel gently to 140 °C until all moisture evaporated. The process flow diagram showing these steps is given in Fig. 1. The fuel properties of pure diesel and algae biodiesel were evaluated using various instruments, with all recorded values presented in Table 1.

**Table 1:** Fuel properties comparison of pure diesel, biodiesel with its blends.

FUEL PROPERTIES	UNITS	ASTM STANDARDS	D 100	Braunii Algae Oil	A1OO	A30
**Density at (20** ^**o**^ **C)**	kg/m^3^	D1298	820	950	920	*815*
**Kinematic viscosity at 40** ^**o**^ **C**	mm^2^/s	D445	3.12	16.45	*6.91*	*4.62*
**Flash point**	oC	D93	63	150	*125*	*51*
**Fire point**	oC	D93	72	160	*133*	*56*
**Calorific value**	kJ/kg	D240	43,581	40,000	*43,280*	*42,381*
**Cetane Index**	–	D4737	49	50	58	53

### FTIR fuel charcteristics

**Fig. 2:**
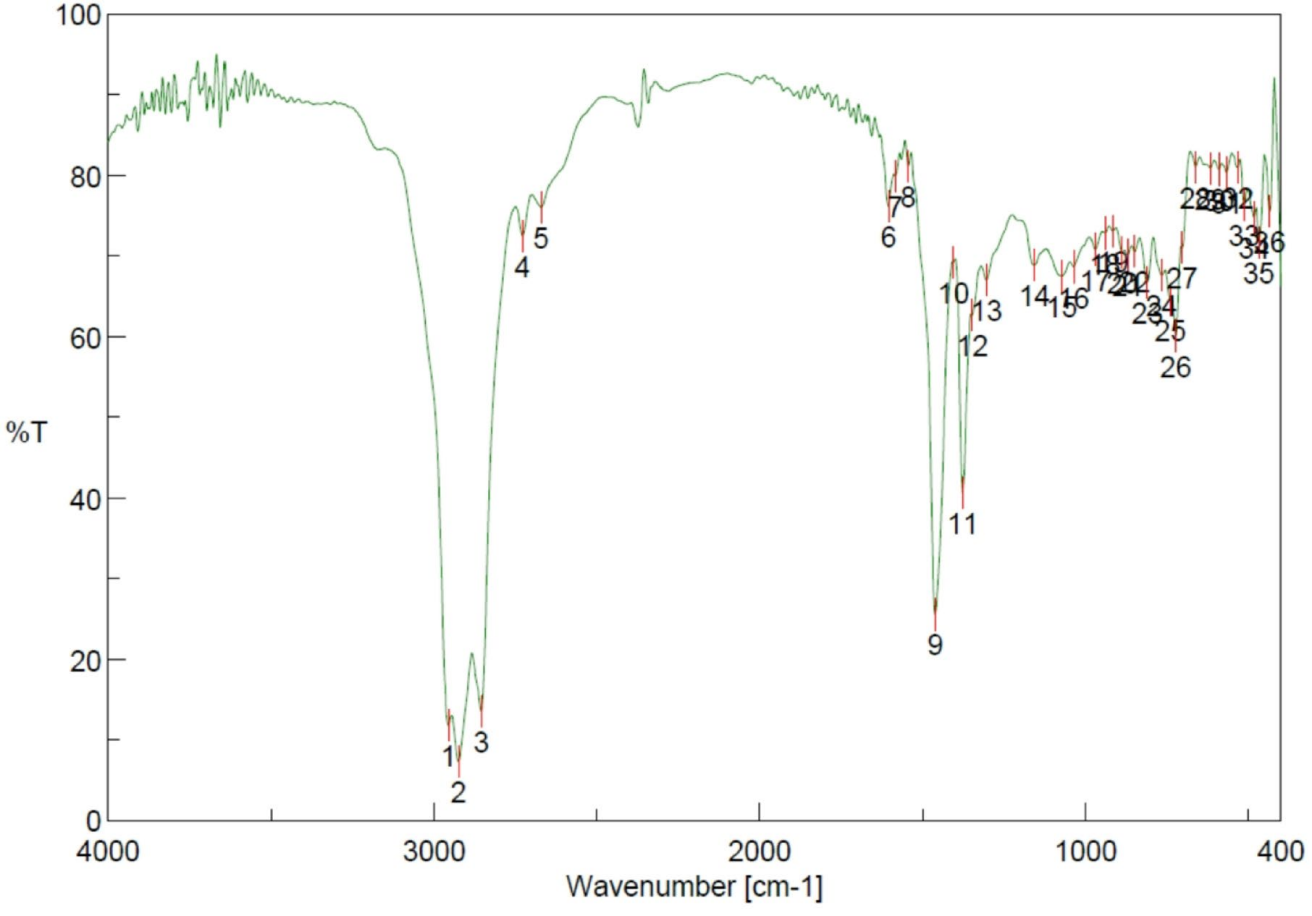
FTIR analysis in the spectrum D100 (Pure Diesel).

**Fig. 3:**
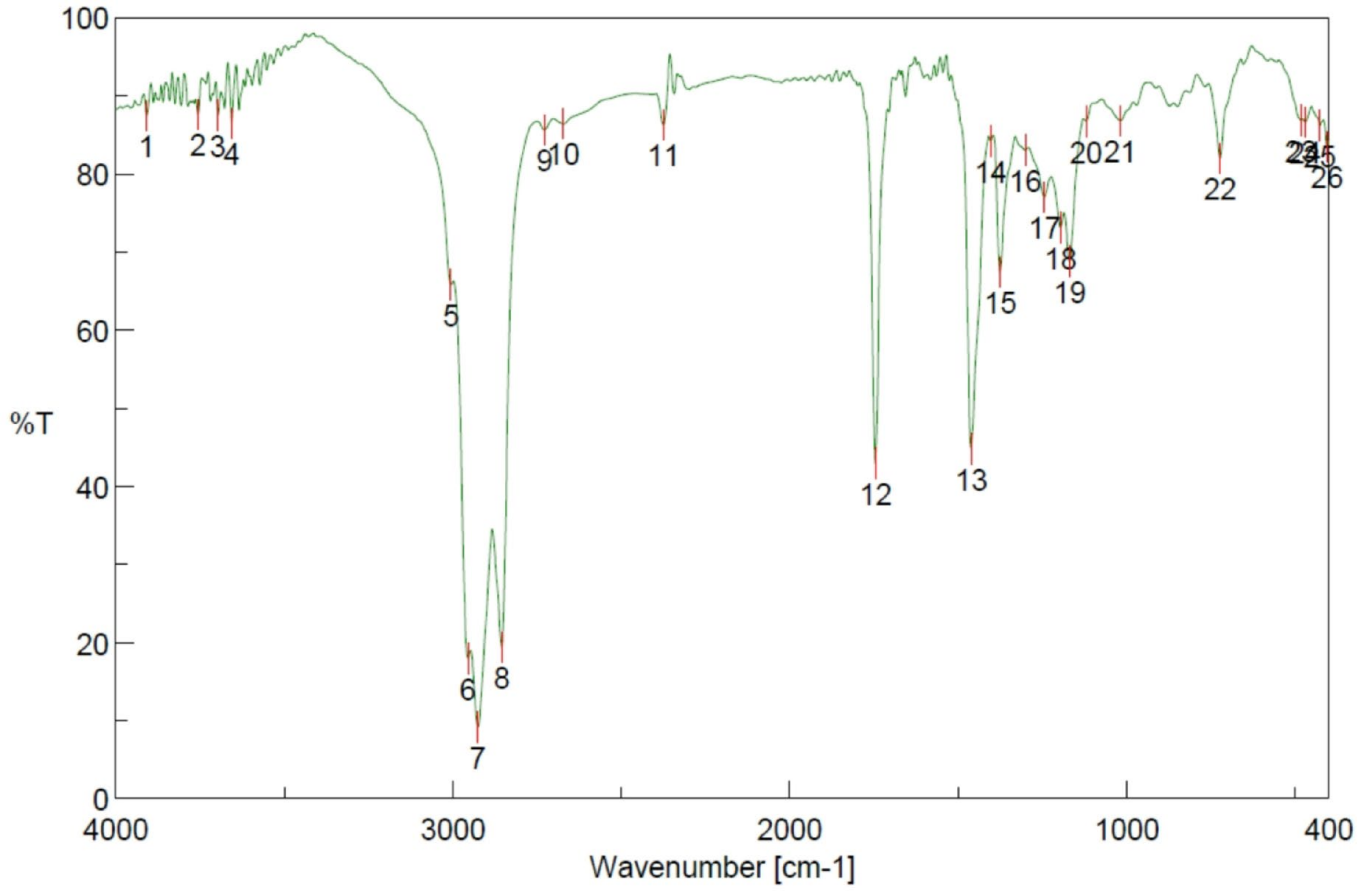
FTIR analysis in the spectrum A30 (70% diesel and 30% Botryococcus braunii algae biodiesel).

The Fourier Transform Infrared (FTIR) spectroscopy investigates the molecular structures and the composition of the two fuels, viz., D100 and A30 is shown in Figs. 2 and 3. It portrays the differences between the two, in terms of its structural composition and chemical composition. The key absorption bands occur at 2855–2924 cm⁻¹ and 1461.78 cm⁻¹ respectively which prominently refer to the alkanes with C-H bond vibrations. The minor peak is witnessed at 723.17 cm⁻¹. The signs indicate that the stronger peak is due to the presence of C-H atoms and the weak sign indicates that it consists of hydrocarbons. Contrary to the above, A30 observes an absorption band at 1745.26 cm⁻¹, showing a corresponding C-O vibration of esters and aldehydes. Nevertheless, this peak is not observed in D100 due to the existence of Fatty Acid Methyl Esters (FAMES). The larger band of C-H, ranging between (2855–2955 cm⁻¹) and sharper ester bands cite enhanced and efficient bonding of the molecules alongside a rich content of hydrogen and effective reactivity. Thus, A30 is inferred that the FITR profile demonstrates enhanced and better vibrations along with top-notch quality of fuel, in comparison to D100.

### Experimental setup

**Fig. 4:**
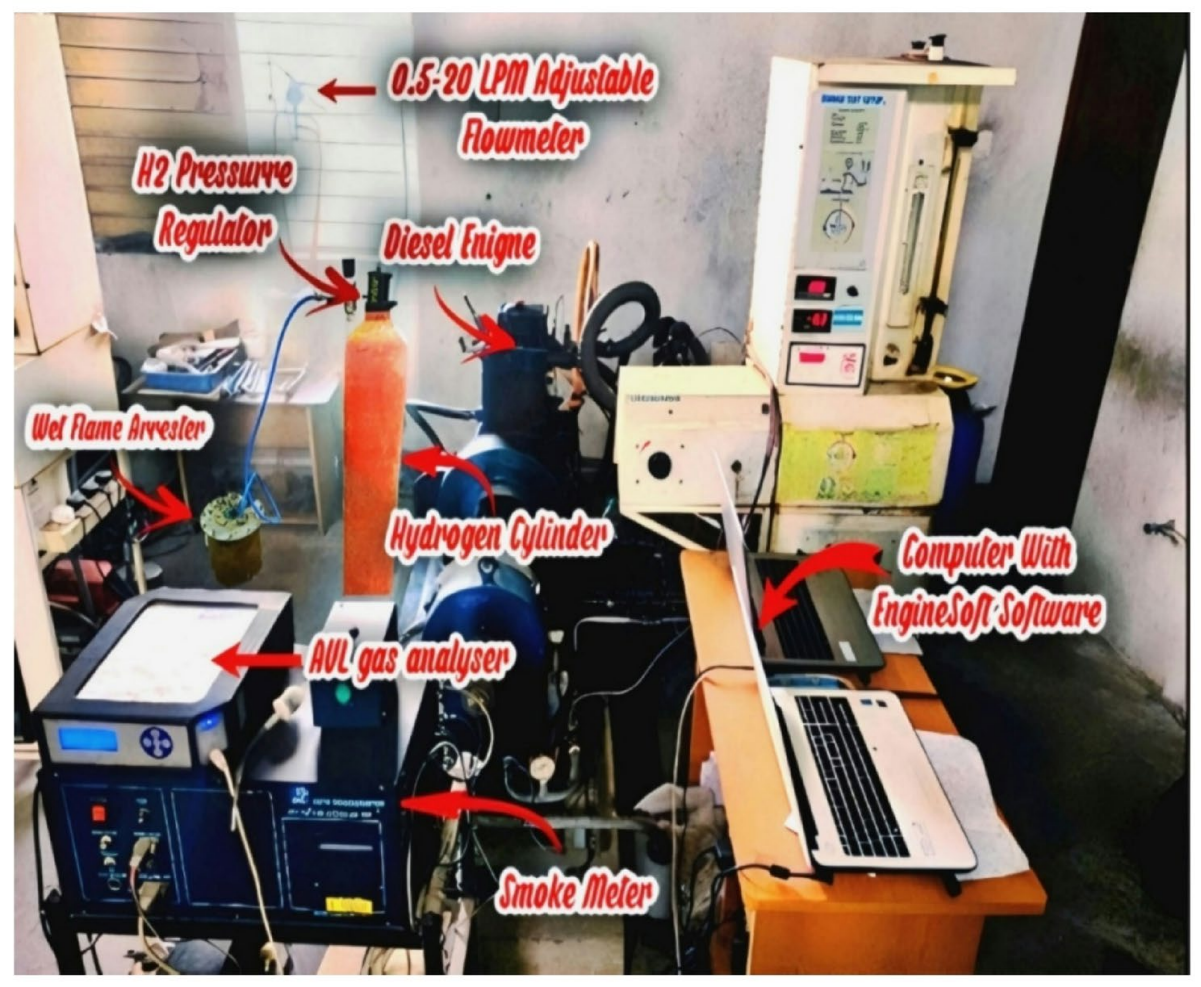
Engine Setup.

**Fig. 5:**
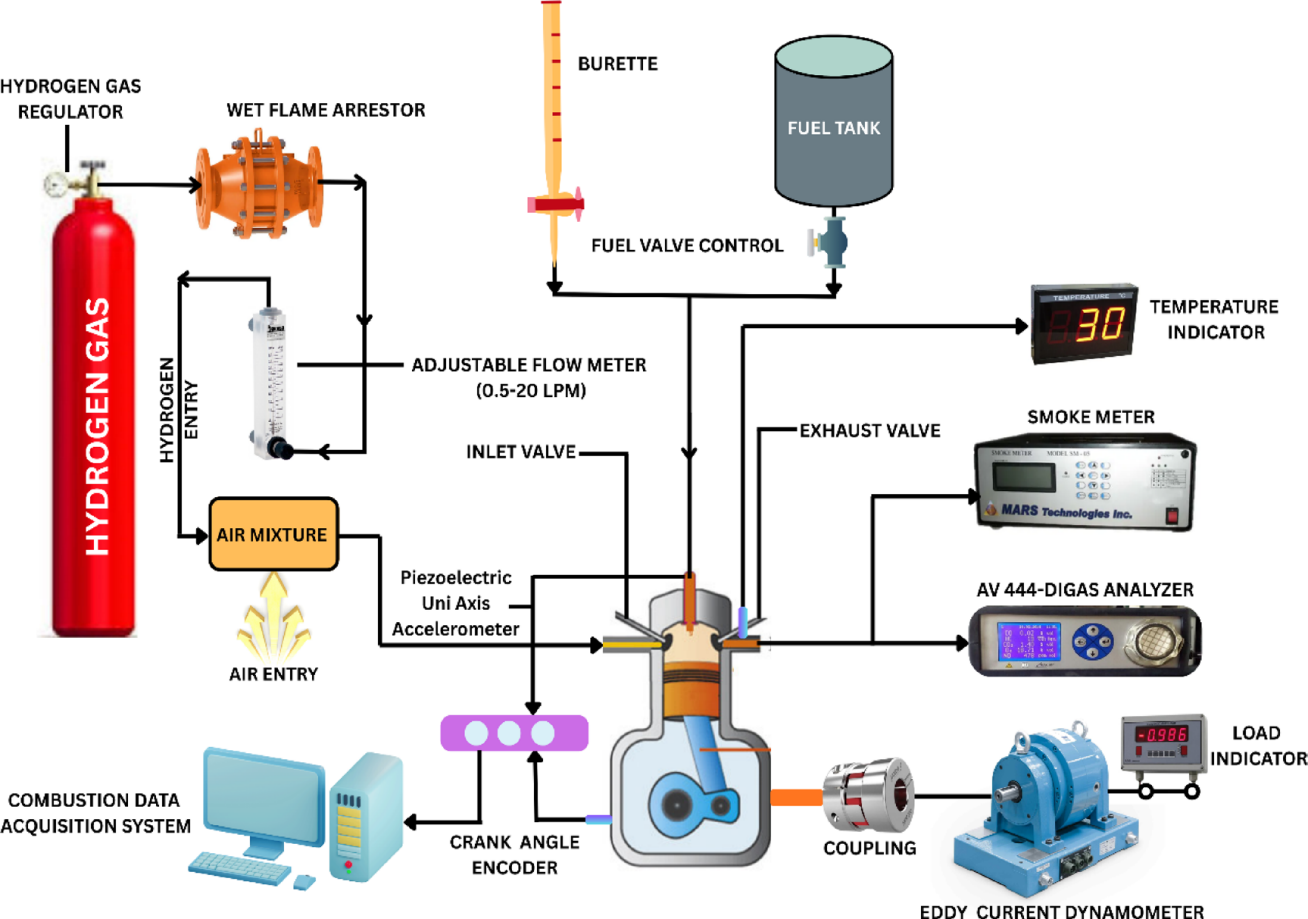
Engine layout.

The researchers constructed a Kirloskar TV1 single-cylinder direct-injection four-stroke diesel engine test rig that functions as the main experimental platform for biodiesel-hydrogen fuel blend performance testing and emission measurements. The 1500 rpm engine uses 87.5 mm for bore size and 110 mm for stroke length to produce a 661 cc displacement volume. Water cooling acts as the main method of measurement because it enables exact readings through the eddy current dynamometer connection. A storage system employing hydrogen operates fuel injection safely and optimally. Figure 4 shows the complete experimental setup that contains all research study components and engine specifications shown in Table 2. For fire safety protection the high-pressure hydrogen cylinder contains both flame trappers and arresters. The solenoid-based electronic injector that utilizes five nozzles with 0.126 mm diameter determines hydrogen injection rate with help from the mass flow meter system. Through pulse-width modulation (PWM) controlled by the Atmega-328 microcontroller timing of hydrogen injection is activated to manage flow according to engine parameters. The L293D adapter distributes power supply from the 5-bar hydrogen pump through a microcontroller to the injector unit. The engine receives hydrogen at different crank angles during the intake phase beginning at 70.3°CA followed by 140.6°CA and concluding with 210.9°CA in order to enhance combustion performance. Figure 5 illustrates through a visual representation the main devices involved in the engine operation. Hydrogen safety protocols included an MQ-8 leak detector (sensitivity range: 100–10,000 ppm) installed near the injector line, SS-316 hydrogen piping, certified flame-arrestor protection, and dual leak-checking procedures using soap-bubble testing and electronic monitoring to ensure safe operation. The hydrogen system used a two-stage YR-72 regulator (0–200 bar), a 150-bar relief valve, a fast-response 12 V solenoid valve and SS-316 piping to ensure stable flow, pressure safety, and leak-free hydrogen delivery.

**Table 2:** Engine experimental setup and specification.

Engine parameter	Specification
Product	CRDI Engine
ECU	Model Nira i7r with programmable ECU software
Engine make	Kirloskar, Single cylinder, 4 stroke.
brake power	3.5 KW @1500 rpm
Bore x stroke, displacement	87.5 × 110 mm, 661 cc
Number of nozzle hole and diameter	3 holes and 0.3 mm
Injection timing	23^o^bTDC
Injection pressure	600 bar
Compression ratio	17.5:1
Category of cooling and ignition system	water cooled and CI
Dynamometer	Eddy current type, water cooled with the loading unit
Data acquisition device	NI USB-6210, 16-bit, 250kS/s.
Hydrogen gas flow meter	Alicat Scientific M-Series; range: 0.5–20 LPM;
	Accuracy: ±0.8%.
Hydrogen Injector	Brand: Bosch Electronic Gaseous Injector
	Nozzle dia: 0.126 mm
	Pressure: 5 bar
	5-hole pattern
	Injector location: mounted on intake manifold

The collection of real-time combustion pressure data along with fuel flow amount and exhaust gases took place through “Enginesoft” software. A dual analysis occurred through AVL DI GAS 444 N for CO, CO_2_, Nox and HC and O_2_ emissions while AVL 437 C Smoke Meter evaluated particulate matter is shown in Table 3. The research examined biodiesel blends A30 and 100% Botryococcus braunii biodiesel (A100) and D100 fuel under two different hydrogen enrichment levels set at 4 LPM and 8 LPM. The hydrogen fuel properties shown in Table 4. The controlled fuel delivery system in Figs. 4 and 5 featured a flashback arrester and bubbler for hydrogen supply to the engine. Experimental results demonstrated higher fuel efficiency alongside lower exhaust emissions which validated hydrogens potential as an extra fuel addition. Direct-injection diesel engines can benefit from biodiesel-hydrogen blended fuels which serve as an appropriate answer to minimize greenhouse gas air pollutants.

**Table 3:** Exhaust gas emission analyzer measurement data and resolution.

Measurement data	Resolution
HC – 0-20000ppm Vol	1ppm/10ppm
CO2 −0-20% Vol	0.1% Vol
O2–0–25% Vol	0.01% Vol
CO −0-15% Vol	0.0001% Vol
NOx − 0-6000ppm Vol	1ppm Vol
Opacity − 0–100%	0.10%
Absorption (K Value)	0–99-99 m^− 1^ 0.01 m^− 1^

**Table 4:** Physiochemical properties of H_2_.

PROPERTIES	ASTM	H_2_
Octane number	D 2699	136
Density, kg/m^3^	D 3588	0.089
Autoignition Temperature, ℃	-	623
CV, MJ/kg	D 1945	146.37
Flashpoint, ℃	-	−254
Fire point, ℃	-	−245
Purity, %	D 7941	99.99

### Uncertainty analysis

The uncertainty analysis of the entire experiment was calculated using this equation.

The combined uncertainty (π) is calculated using the root sum square method:

$$\begin{aligned} \pi= \:\surd\:\left[\right(\mathrm{C}\mathrm{O}_2)^2\:+\:(\mathrm{C}\mathrm{O})^2\:+\:(\mathrm{O}_2)^2\:+\:(\mathrm{H}\mathrm{C})^2\:+\:(\mathrm{N}\mathrm{O})^2\:+\:(\mathrm{B}\mathrm{T}\mathrm{E})^2\:+\:(\mathrm{S}\mathrm{F}\mathrm{C})^2\:+\:(\mathrm{N}\mathrm{H}\mathrm{R}\mathrm{R})^2\:\\+\:(\mathrm{s}\mathrm{m}\mathrm{o}\mathrm{k}\mathrm{e})^2\:+\:(\mathrm{t}\mathrm{e}\mathrm{m}\mathrm{p}\mathrm{e}\mathrm{r}\mathrm{a}\mathrm{t}\mathrm{u}\mathrm{r}\mathrm{e})^2\:+\:(\mathrm{p}\mathrm{r}\mathrm{e}\mathrm{s}\mathrm{s}\mathrm{u}\mathrm{r}\mathrm{e})^2\:+\:(\mathrm{l}\mathrm{o}\mathrm{a}\mathrm{d})^2\:+\:(\mathrm{c}\mathrm{r}\mathrm{a}\mathrm{n}\mathrm{k}\:\mathrm{e}\mathrm{n}\mathrm{c}\mathrm{o}\mathrm{d}\mathrm{e}\mathrm{r})^2]\end{aligned}$$

Substituting the given values:

$$\begin{aligned} \pi \:=\:\surd\:\:\left[\right(2.0)^2\:+\:(2.0)^2\:+\:(1.0)^2\:+\:(0.2)^2\:+\:(0.2)^2\:+\:(0.15)^2\:+\:(0.15)^2\:+\:(0.2)^2\:\\+\:(1.0)^2\:+\:(0.5)^2\:+\:(1.0)^2\:+\:(0.2)^2\:+\:(0.2)^2\:+\:(0.2)^2]\end{aligned}$$

$$\:\:=\:\surd\:\:\left(11.535\right)\hspace{0.17em}\approx\:\hspace{0.17em}3.40$$

## Result and discussion

### Performance characteristics

#### Brake thermal efficiency

**Fig. 6:**
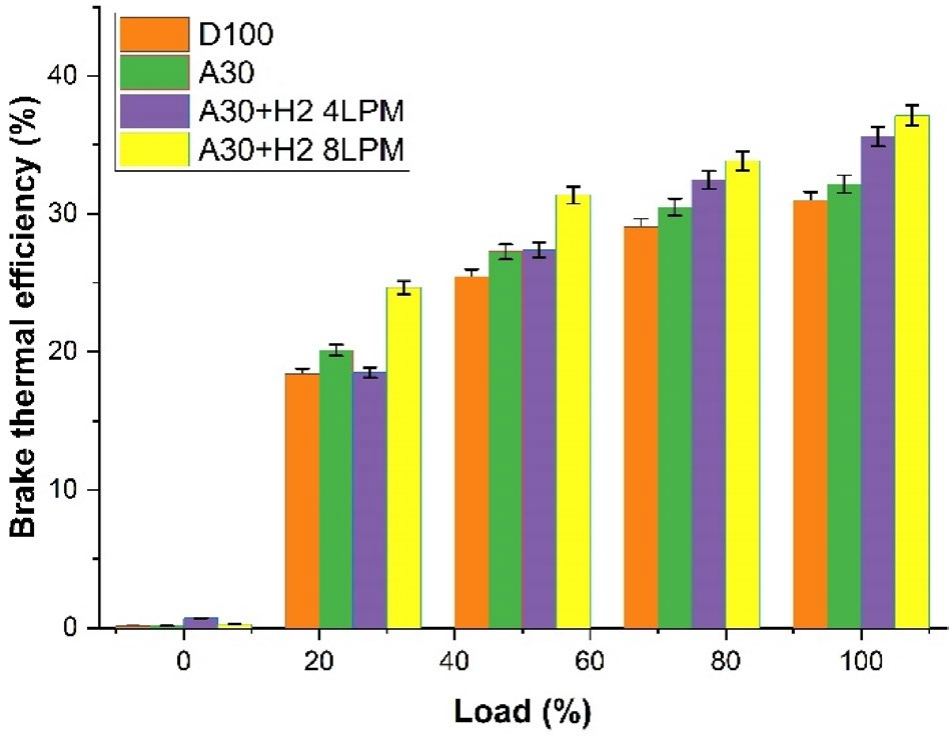
– Load Vs Brake Thermal Efficiency (%).

The Fig. 6 shown the effect of the engine load on the brake thermal efficiency for the four different fuels, namely, D100, A30, A30 + H_2_ (4 LPM) and A30 + H_2_ (8 LPM). The results indicate that the BTE is considerably increased as the engine load increases for all the fuels. This is due to the fact that the increasing loads enhances better combustion pressure, resulting in lower heat loss and effective use of the fuel. It is much evident from the graph that the blend A30 + H_2_ (8 LPM), exhibits the maximum BTE across the complete operating range. It is noted that at the peak load, the value of BTE is 31% relative to D100, 32% for A30, a surge of 3% in comparison to D100, 35% for the blend A30 + H_2_ (4 LPM), indicating a 13% progress over D100 and 19% for the blend A30 + H_2_ (8 LPM), illustrating a 19% growth over D100. The presence of oxygen and the oxygenated characteristics of Botryococcus braunii biodiesel in A30 enhances the quality of combustion in comparison to pure diesel. The addition of increased levels of hydrogen enhances faster combustion, leads to better mixture of the air and the fuel and finally facilitates a superior clean and a better burn of the fuel, thereby resulting in improved thermal efficiency at the highest load.

### Specific fuel consumption

**Fig. 7:**
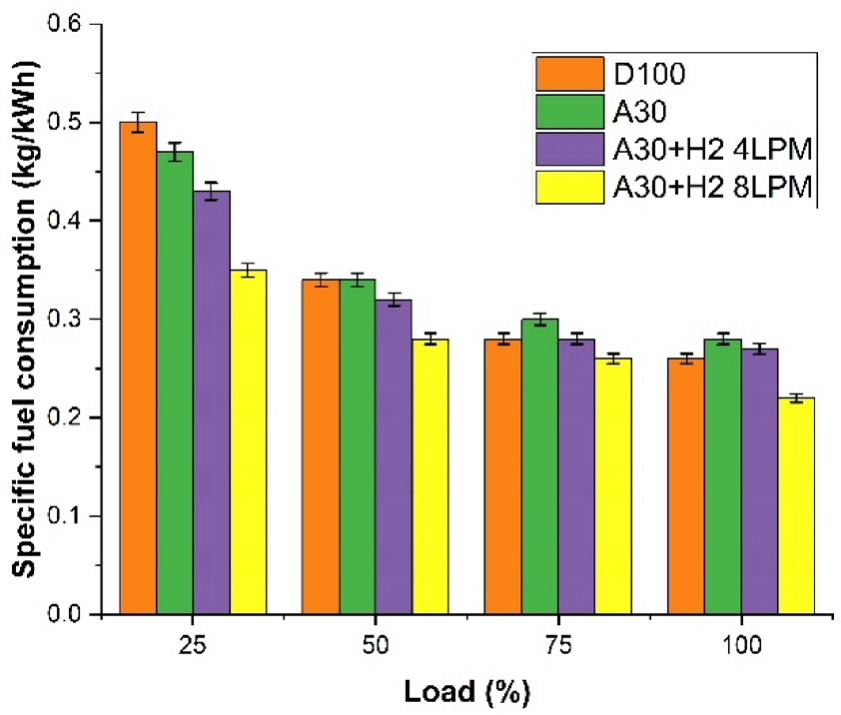
Load Vs Specific Fuel Consumption (kg/kWh).

The Fig. 7 graphical representation depicts how the specific fuel consumption varies with the engine load for the four different fuels, viz., D100, A30, A30 + H_2_ (4 LPM), and A30 + H_2_ (8 LPM). It is observed that as the engine load increases, the SFC constantly decreases for all the four fuels. This is due to the fact that the increasing loads leads to increased combustion temperatures, enhanced pressure of the cylinder resulting in highest use of the fuel, enhancing the engine to extract the highest potential output of power. It is much evident from the graph that the blend A30 + H_2_ (8 LPM), exhibits the minimum SFC across the fullest operating load conditions. It is noted that at the peak load, the values of SFC is 0.30 kg/kWh for D100, 0.29 kg/kWh for A30, a decline of 3% in comparison to D100, 0.27 kg/kWh for the blend A30 + H_2_ (4 LPM), indicating a drop of 10% over D100 and 0.24 kg/kWh for the blend A30 + H_2_ (8 LPM), illustrating a fall of 20% over D100. The presence of oxygen and the oxygenated characteristics of Botryococcus braunii biodiesel in A30 enhances cleaner and complete combustion of the fuel. The addition of increased levels of hydrogen enhances faster combustion, reduces and lessens the unburnt fuel, leading to better mixture of the air and the fuel and finally facilitates a superior fuel economy with clean and a better burn of the fuel.

### Volumetric efficiency

**Fig. 8:**
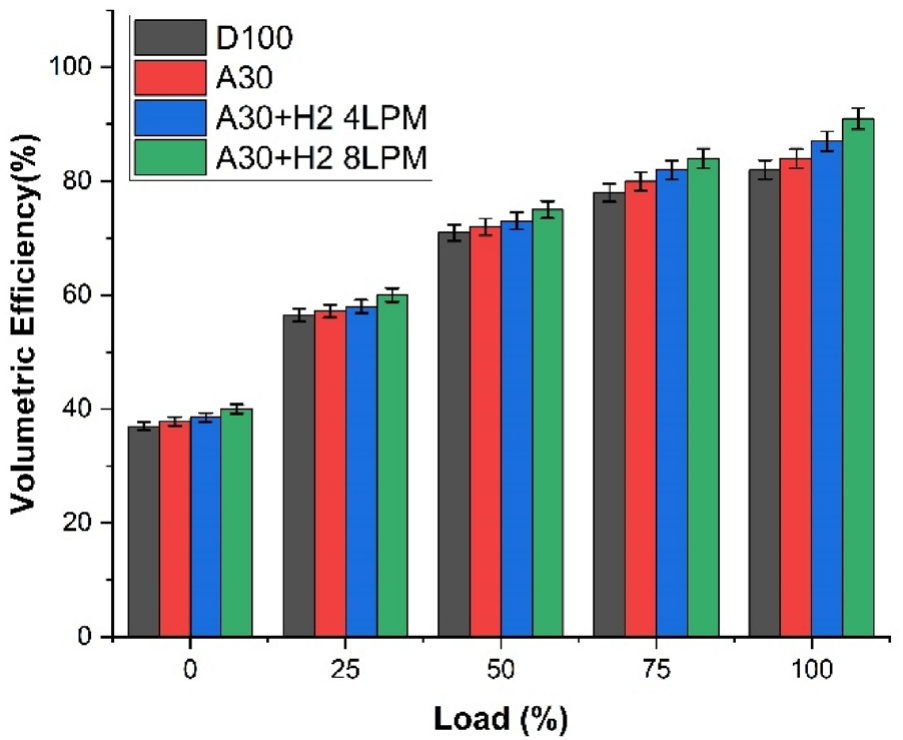
– Load Vs Volumetric efficiency.

The volumetric efficiency is calculated using Eq. (1), which expresses the ratio of the actual mass flow rate of air inducted into the engine to the theoretical air capacity determined by air density, engine displacement volume, and engine speed for a four-stroke engine. The Fig. 8 graphical representation shows the analysis of volumetric efficiency of Botryococcus braunii biodiesel with hydrogen blends under various load conditions for the four different fuels, viz., D100, A30, A30 + H_2_ (4 LPM), and A30 + H_2_ (8 LPM). It is observed that as the engine load increases, the volumetric efficiency significantly increases for all the four fuels. This is caused due to the higher consumption of air, which increases the suction pressure and decreases the pumping losses drastically. From the results generated, it is evident that the volumetric efficiency is lower for D100 while it is much higher for the biodiesel with hydrogen blends. It is noted that at the peak load, the volumetric efficiency is 82% for D100, 84% for A30, a surge of 2.4% in comparison to D100, 87% for the blend A30 + H_2_ (4 LPM), indicating a 6.1% progress over D100 and 91% for the blend A30 + H_2_ (8 LPM), illustrating a 11% growth over D100. The presence of oxygenated characteristics of algae biodiesel in A30 reduces the residual gases, thus enhancing a very efficient and a clean combustion, permitting an effective induction of air. The fast and high flame velocity of hydrogen improves the stability of combustion to a greater extent, facilitating better breathing capacity and capability of air intake of the engine. It is notably seen that the blend A30 + H_2_ (8 LPM), allows a very cleaner and quicker combustion, diminishing the effects of back-pressure, reducing the quantum of exhaust gases, resulting in a greater filling of the cylinder. In a nutshell, it is much predominant that Botryococcus braunii biodiesel with hydrogen enrichment substantially increase the volumetric efficiency compared to the pure diesel.

1 $$\:{\eta\:}_{v}=\frac{{\dot m}_{air}}{{\rho\:}_{air}\times\:\:{V}_{d}\:X\frac{N}{2}\:}$$

Where:

η_v_ = Volumetric efficiency.

ṁ_air_ = Actual mass flow rate of air inducted into the engine (kg/s).

ρ_air_ = Density of air at intake conditions (kg/m³).

V_d_ = Engine displacement volume (m³).

N = Engine speed (rpm).

N/2 = Number of intake strokes per minute for a four-stroke engine.

### Combustion characteristics

#### Cylinder pressure

**Fig. 9:**
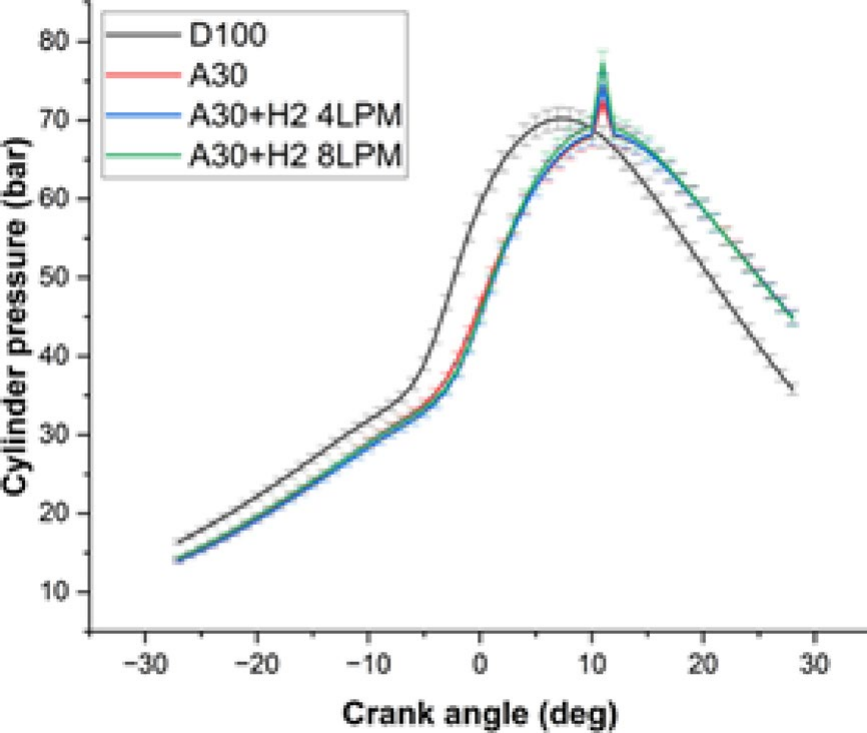
– Crank angle Vs Cylinder pressure.

The study examined how the pressure inside the diesel engine for the Botryococcus braunii biodiesel with hydrogen blend was affected for different types of fuels is revealed in Fig. 9. The analysis was carried out with four different fuels and at the maximum load capacity. The fuels which were investigated for the study were D100, A30, A30 + H_2_ (4 LPM) and A30 + H_2_ (8 LPM). Owing to quick swift of the combustion process, it was observed that all the fuels display a characteristic and similar combustion pattern, resulting in rapid increase of the cylinder pressure in the vicinity of the Top Dead Center (TDC). Nevertheless, the pressure was found to vary with various composition of fuels at the maximum load pressure. It was observed that pure diesel displayed a n highest pressure of 70.2 bar whereas A30 bend demonstrated the maximum of 72.4 bar, a surge of around 3.1%. This spike is because of the availability of the oxygen in A30, turning it to burn more seamlessly and effectively and facilitating for additional decrease in ignition lag. The addition of hydrogen at 4LPM to the blend, A30 + H_2_ (4 LPM) increases the pressure to 74.4 bar, which is 6% higher than D100. This added hydrogen allows the fuel to burn better and due to the increase in pressure, it facilitates for the smooth mixing with air. The best results were depicted with A30 + H_2_ (8 LPM) reaching the highest and maximum of pressure of 77.3 bar, a 10.1% increase relative to D100. The above results clearly portrays that more hydrogen content tends to combust the fuel more efficiently in the vicinity of TDC. On the whole, it is obviously noticeable that Botryococcus braunii biodiesel with hydrogen blends enhances for better combustion and establishing it to be a superior alternative than the pure diesel.

### Net heat release rate

**Fig. 10:**
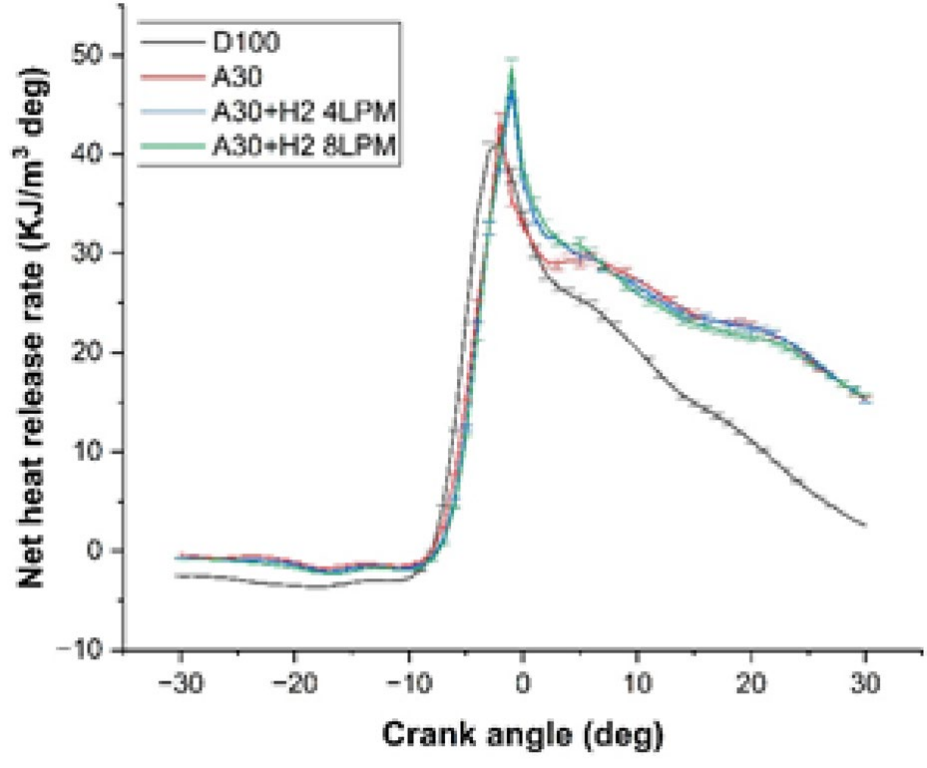
Crank Angle Vs Net Heat Release Rate.

The study demonstrated the examination of the heat release rate for the Botryococcus braunii biodiesel with hydrogen blend exposed in Fig. 10. It was clearly seen that it was affected for different types of fuels. The analysis was carried out with four different fuels and at the maximum load capacity. The fuels which were investigated for the study were D100, A30, A30 + H_2_ (4 LPM) and A30 + H_2_ (8 LPM). Heat release rate shows how quickly the energy is released during combustion. Owing to quick swift of the Heat release rate, it was observed that all the fuels display a characteristic and similar combustion pattern, resulting in rapid increase of the heat release rate in the vicinity of the Top Dead Center (TDC). Nevertheless, the values of NHRR significantly varies with the composition of fuel. The results showed that the peak NHRR was 41.11 kJ/m^3^deg for D100 and 43.28 kJ/m^3^deg for A30, marking an increase of about 5.3% compared to pure diesel. This surge is occurred due to the availability of oxygen in the algae-based biodiesel molecular composition that enhances and boosts combustion. The analysis further extends by incorporating hydrogen at 4LPM, the blend A30 + H_2_ (4 LPM) exhibits a peak NHRR of 46.45 kJ/m^3^·deg, which is 13% increase in comparison with pure diesel. This increase is because of the rapid flow of flame of hydrogen, leading to better mixing of fuel in the air. Among all the type of fuels, A30 + H_2_ (8 LPM) shows the peak NHRR of 48.63 kJ/m^3^deg, illustrating a rise of 18.3% in comparison with pure diesel. This obviously shows that added content of hydrogen to the biodiesel, facilitates quicker and more effective combustion, harnessing more energy in the vicinity of the top dead center (TDC). In nutshell, it is evident that adding hydrogen to Botryococcus braunii biodiesel, enhances and effectuates combustion efficiency, as opposed to pure diesel.

### Emission characteristics

#### Carbon monoxide emission

**Fig. 11:**
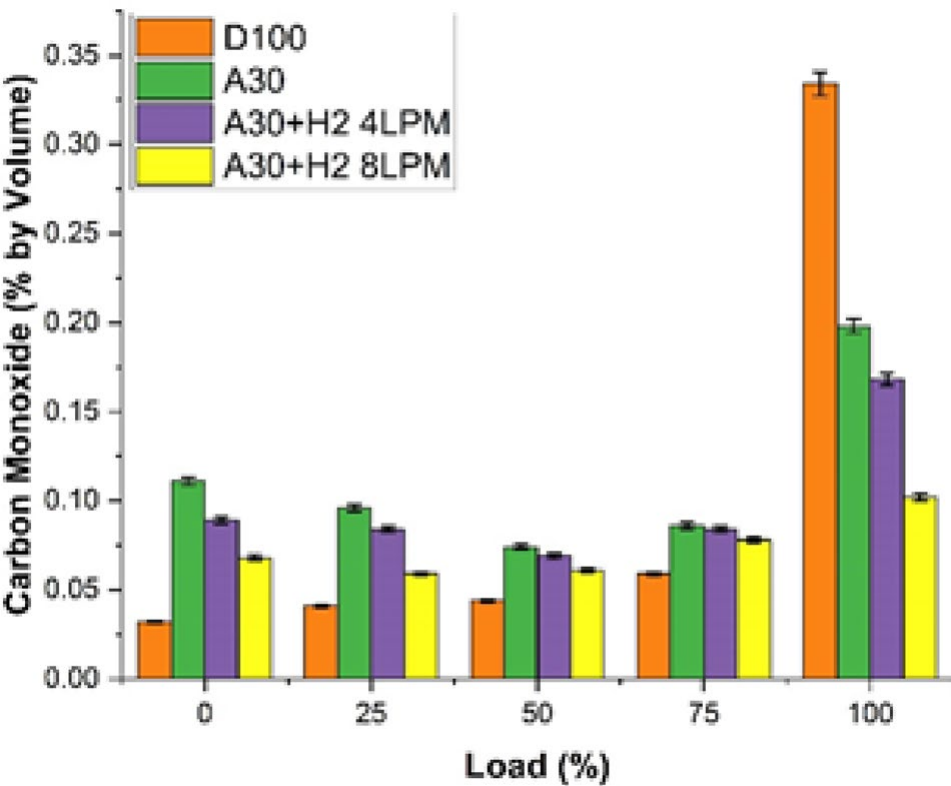
Load vs. Carbon Monoxide.

The study examined and explored the characteristics of the carbon monoxide emissions of biodiesel produced from Botryococcus braunii algae enriched with blends of hydrogen is shown in Fig. 11. The analysis was carried out with four different fuels and at the maximum load capacity. The fuels which were investigated for the study were D100, A30, A30 + H_2_ (4 LPM) and A30 + H_2_ (8 LPM). It is observed that as the engine load increases, the CO emissions also simultaneously increased for all the fuels due to the fuel-rich combustion and lower oxygen levels. On the other hand, it was also identified that hydrogen blend with A30 emitted lower CO compared to the conventional type of diesel engine. This is due to the blends’ improved oxidation potential, responsible for the same. It was observed that at the maximum load, D100 emitted CO of 0.334%, which was the maximum among all the fuels tested. In contrast to it, the CO emissions emitted by A30 was 0.198%, illustrating a 40.7% lower than the pure diesel. This is because of the availability of oxygen in the algae biodiesel, facilitates to the full transformation of CO-to-CO_2_. In view of the above. Further addition of hydrogen leads to decreased emissions of CO. The A30 + H₂ (4 LPM) emits 0.168% CO, indicating a drop of 49.7% in comparison with pure diesel. Furthermore, A30 + H_2_ (8 LPM) emits the lowest among all fuel types, yielding 0.102% indicating a substantial drop oof 69.5% in comparison with pure diesel. With the flow rate of hydrogen being at high levels, exhibiting a very clean and better combustion, thus minimising the unburned fuel zones. In brief, the essence of the results demonstrates that algae biodiesel with hydrogen, considerably reduces the CO emissions compared to the pure diesel, rendering it as an eco-friendlier and an alternative fuel option.

**Fig. 12:**
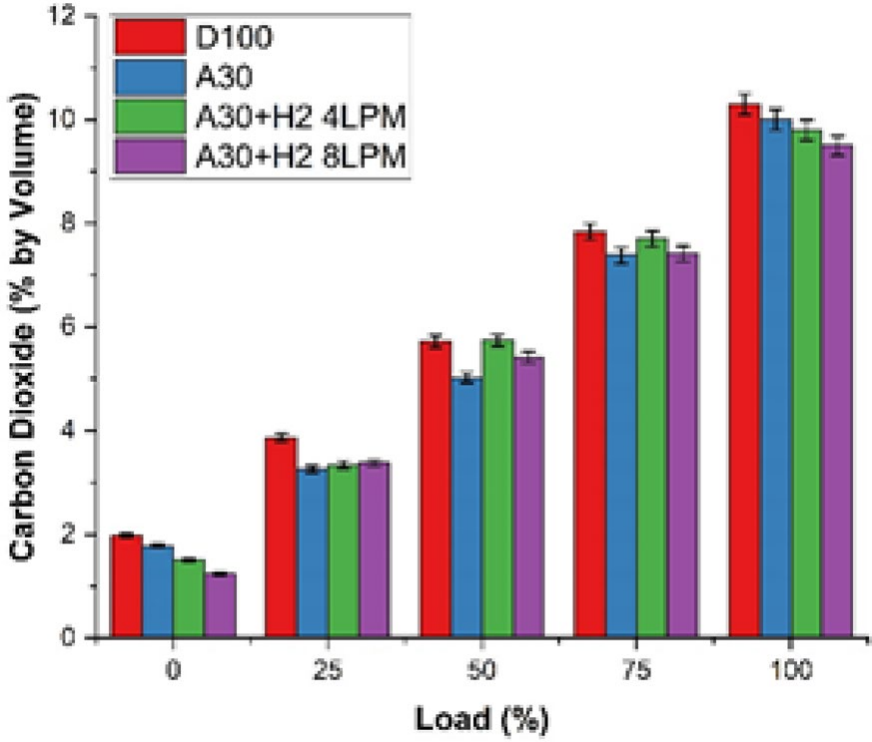
Load vs. Carbon dioxide.

### Carbon dioxide emission

The study examined and explored the characteristics of the carbon dioxide emission profile of biodiesel produced from Botryococcus braunii algae enriched with blends of hydrogen is shown in Fig. 12. The analysis was carried out with four different fuels and at the maximum load capacity. The fuels which were investigated for the study were D100, A30, A30 + H_2_ (4 LPM) and A30 + H_2_ (8 LPM). It is observed that as the engine load increases, the CO₂ emissions also concurrently increased for all the fuels. This occurs due to higher consumption of fuel and elevated combustion temperature at heavier loads. It was observed that at the maximum load, D100 emitted CO_2_ of 10.3%, which was the maximum among all the fuels tested. In contrast to it, the CO_2_ emissions emitted by A30 was at 10% which is 2.9% lesser than D100. This is due to the fact that the algae biodiesel in A30 has high oxygen and less carbon content, facilitating the fuel to burn fully. As indicated earlier, the addition of hydrogen in A30 blend brings down the CO_2_ emissions drastically. Adding hydrogen to A30, (A30 + H_2_ 4 LPM) brings down the CO₂ emissions to 9.8% which is 4.9% lesser than D100. Further on addition of hydrogen (A30 + H_2_ 8 LPM) to A30, drops further to 9.5% which is 7.8% under D100. It is understood that the addition of more hydrogen to the blend of A30, initiates a rapid flow of the flame with higher efficiency and as the engine depends less on the fuel molecules containing carbon, the engine generates less CO_2_, as only a small quantum of carbon stands available to turn into CO_2_. The above results concisely demonstrates that Botryococcus braunii biodiesel with hydrogen emits less CO_2_, validating as significant and strong potential for a cleaner and a low-carbon generated alternative fuel, that could replace diesel.

### Hydrocarbon emission

**Fig. 13:**
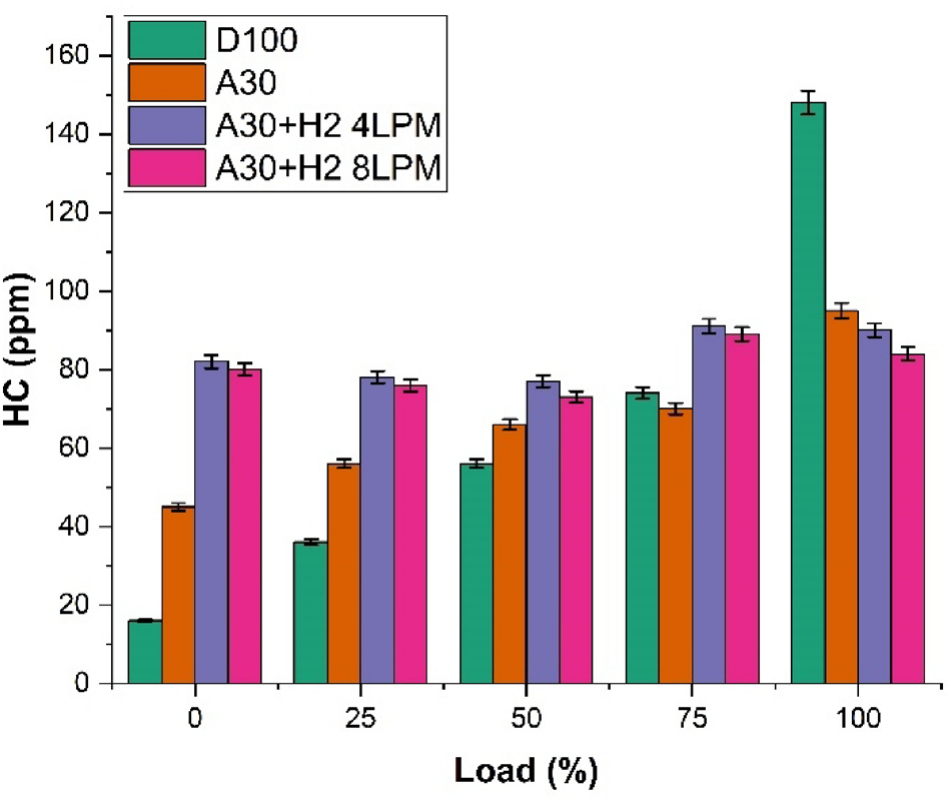
Load vs. Hydrocarbon Emission.

The study investigated the characteristics of the hydrocarbon emission profile of biodiesel produced from Botryococcus braunii algae enriched with blends of hydrogen is presented in Fig. 13. The analysis was carried out with four different fuels and at the maximum load capacity. The fuels which were investigated for the study were D100, A30, A30 + H_2_ (4 LPM) and A30 + H_2_ (8 LPM). It is observed that as the engine load increases, the CO_2_ emissions also substantially increased for all the fuels. It is due to fuel-rich combustion areas and decreased performance of oxidation. It was noticed that at highest load, D1000 exhibited the utmost HC emissions, recording 148 ppm. It is because of the fact that the diesel fuel doesn’t carry much oxygen in it, which leads to partial and inefficient combustion and resulting in high amount of unburnt fuels in it. The results show that A30 blend exhibits lesser HC emissions (95 ppm), which is 35.8% fall in comparison with D100. It is due to the fact that more oxygen is available in the algae biodiesel, leading to enhanced and efficient oxidation of hydrocarbons. The addition of hydrogen in the algae biodiesel minimizes the HC emissions. As a result of it, A30 + H_2_ (4 LPM) blend emitted lower hydrocarbons (90 ppm), illustrating a 39.2% drop compared to pure diesel. Subsequent to the above, the blend A30 + H_2_ (8 LPM) demonstrates the lowest HC emissions (84 ppm) among all fuel types, which is found to be 43.2% lower than the pure diesel. The addition of more hydrogen facilitates the combustion efficiency with a smooth, cleaner, faster and full burning of the fuel, leading to very lower quantum of unburnt hydrocarbons. The overall investigations conclude that Botryococcus braunii biodiesel with hydrogen lowers the HC emissions drastically, highlighting a superior advantage as opposed to pure diesel.

### Nitrogen oxide emission

**Fig. 14:**
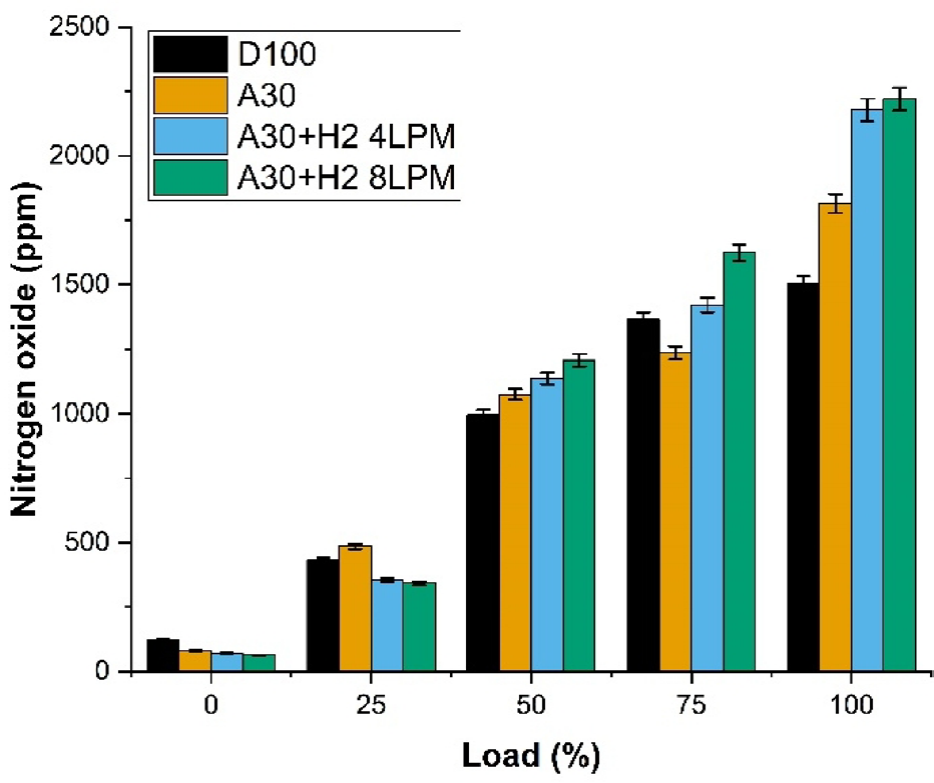
Load vs. Nitrogen Oxide.

The Fig. 14 analysis delved into the characteristics of Nitrogen oxide (NO_X_) of biodiesel produced from Botryococcus braunii algae enriched with blends of hydrogen. The analysis was carried out with four different fuels and at the maximum load capacity. The fuels which were investigated for the study were D100, A30, A30 + H_2_ (4 LPM) and A30 + H_2_ (8 LPM). It is observed that the NO_X_ emissions increase as the burning efficiency increases and it has been noticed that NO_X_ emissions are higher with hydrogen blended biodiesel. It is found that at maximum load, D100 emitted the highest (1503 ppm) NO_X_. A30 emitted 1815 ppm which is around 20.7% higher than D100. This surge is due to the presence of more oxygen in the algae blended biodiesel that supplements the temperatures of combustion and increases the reactions of oxidation rapidly. It is notably seen that addition of hydrogen increases the NO_X_ emissions more. The blend A30 + H_2_ (4 LPM) emits NO_X_ emissions at 2178 ppm, citing an increase of 44.9% above pure diesel. The blend A30 + H_2_ (8 LPM) displays the highest NO_X_ emissions emitted, recording the highest of around 2220 ppm, illustrating an increase of 47.6% against pure diesel. This is because of the fact that hydrogen burns much quicker and its rapid flow makes combustion stronger, leading to the generation of NO_X_ emissions. The results revealed that the incorporation of biodiesel with hydrogen reduces the carbon emissions, but nevertheless it increases the NO_X_ emissions, raising concerns and observed as a potential drawback.

### Smoke opacity emission

**Fig. 15:**
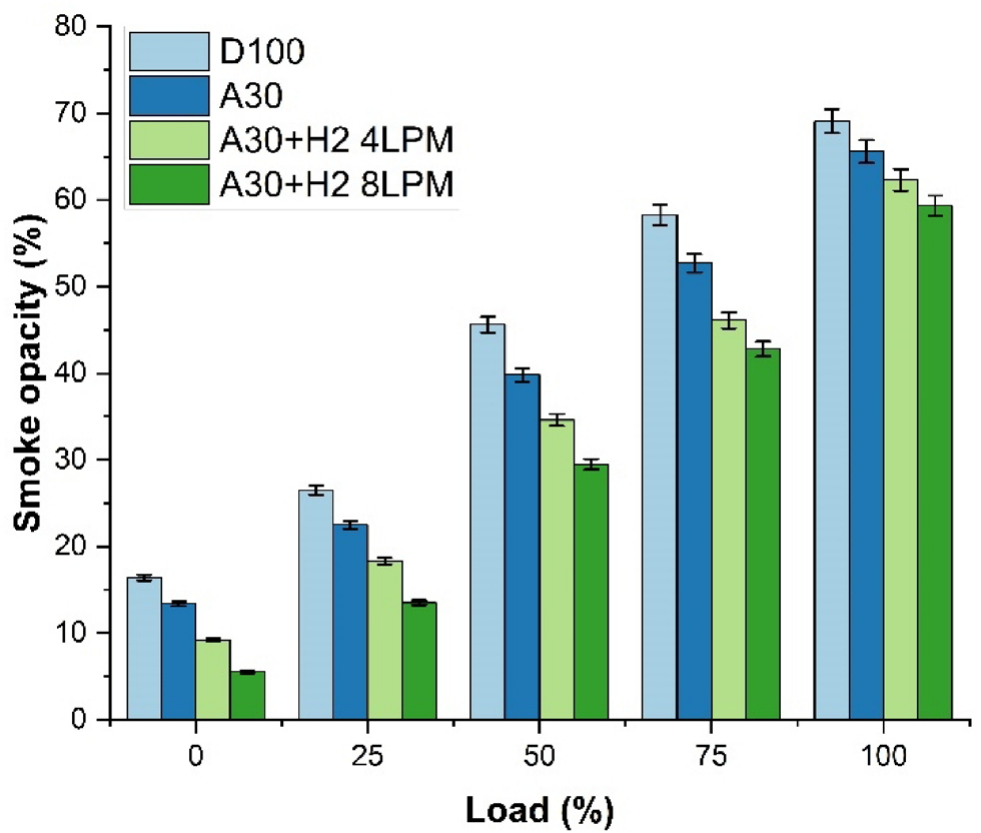
Load Vs Smoke Opacity.

The study analysed the characteristics of smoke opacity of biodiesel produced from Botryococcus braunii algae enriched with blends of hydrogen is shown in Fig. 15. The analysis was carried out with four different fuels and at the maximum load capacity. The fuels which were investigated for the study were D100, A30, A30 + H_2_ (4 LPM) and A30 + H_2_ (8 LPM). It is observed that the smoke opacity concurrently increases with the increase in the engine load. This is due to the consumption of more fuel enhancing fuel-rich combustion with minimized mixture of the fuel with air. The results revealed that the smoke opacity was the highest (69.1%) for D100 at maximum load. This is because of lesser content of oxygen and combustion dominated by diffusion. In contrast to D100, A30 exhibited smoke emissions to the tune of 65.6% illustrating a drop of 5.1% as opposed to D100. This fall is because of the availability of oxygen Botryococcus braunii biodiesel that facilitates the oxidation of soot forming precursors. While, incorporating additional hydrogen to the blend, limits and mitigates the formation of smoke, A30 + H_2_ (4 LPM) blend emits smoke emissions about 62.3% which is 9.8% fall as against to D100. The effect of accelerated and rapid flame progression of hydrogen enhances uniform mixture of the air and the fuel, leading to declination of unburnt regions of carbon. The lowest of all was the A30 + H_2_ (8 LPM) blend with a smoke opacity of 59.3%, indicating a drop of 14.2% over D100. On the whole, algae biodiesel with hydrogen enriched biodiesel are more superior than pure diesel in reducing soot emissions and fosters a cleaner combustion, most efficiently than pure diesel.

**Fig. 16:**
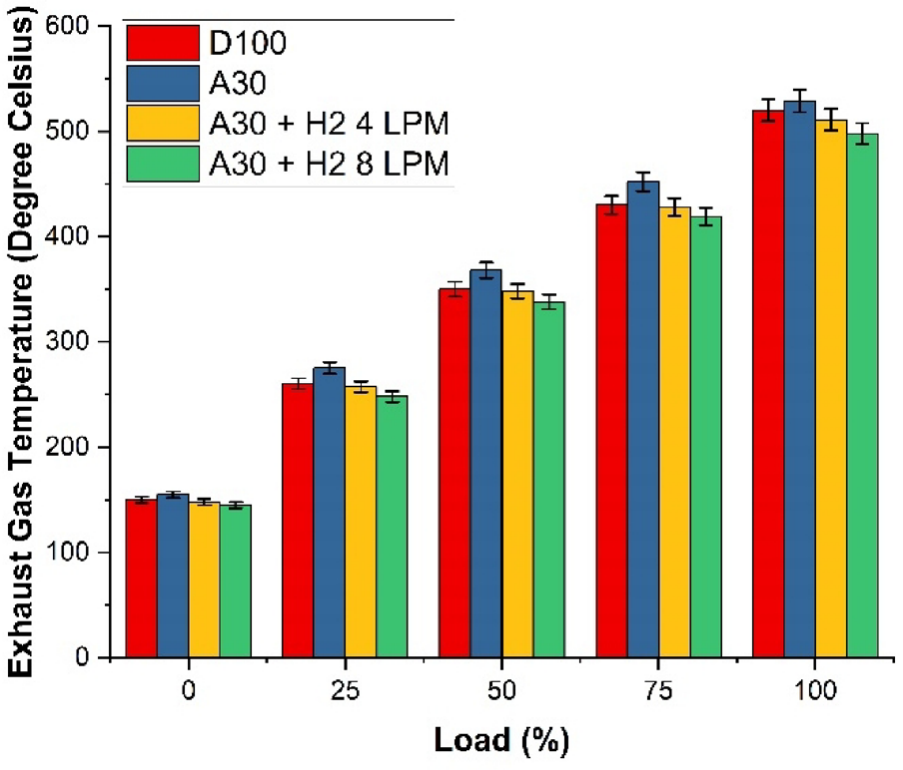
Load Vs Exhust Gas Temperature.

### Exhaust gas temperature (EGT)

The Fig. 16 investigated the features of the exhaust gas temperature of Botryococcus braunii biodiesel with hydrogen. The analysis was carried out with four different fuels and at the maximum load capacity. The fuels which were investigated for the study were D100, A30, A30 + H_2_ (4 LPM) and A30 + H_2_ (8 LPM. It is recorded that the exhaust gas temperature significantly increase for the increase in engine load, for all the four fuels. This is because of the fact that due to excess burning of the fuel, combustion becomes easier and cleaner, resulting in increased combustion temperature. It was also identified that the EGT of A30 was higher than D1-00, due to the availability of more oxygen. The data’s derived shows that D100 has an exhaust gas temperature of 520 °C against 529 °C for A30, illustrating a high rise of 1.73%. This increase is only due to the excess availability of oxygen content in the algae biodiesel and on adding hydrogen content to A30, drastically pulls down the EGT. The blend A30 + H_2_ (4 LPM) shows an EGT of 511 °C, which is 1.73% drop in comparison with D100 and 3.40% lesser than A30. As indicated earlier, the rapid flame propagation of hydrogen yields enhanced characteristics with better combustion leading to very lower level of heat loss in the exhaust. It is noted that A30 + H_2_ (8 LPM) achieves the minimum EGT at 498 °C, indicating a fall of 4.23% with respect to D100 and 5.86% slump than A30. The findings reveal that adding more hydrogen to biodiesel blend augments the effect of combustion, leading to reduction in the leftover heat and subsequently bringing down the exhaust gas temperature.

## Conclusion

The study examined and investigated the overall performance of the engine, characteristics of combustion and the emission patterns of Botryococcus braunii Biodiesel (A30) blended with hydrogen at 2 different rates, vide 4LPM and 8LPM respectively, in comparison with the pure diesel (D100). It was clearly evident that the combination, A30 + H_2_ (8 LPM) stood out and emerged as the most efficient fuel among the samples tested. The tests were much apparent that at the highest load, A30 + H_2_ (8 LPM) resulted in notable and considerable improvements compared to D100. Brake Thermal efficiency (BTE) raised to 37% from 31% due to the blend of A30 + H_2_ 8 LPM in comparison with D100, indicating a rise of 19.35%, but the specific fuel consumption dropped from 0.30 kg/kWh to 0.24 kg/kWh, indicating a fall of 20% over D100. On the contrary, volumetric efficiency showed a substantial progress from 82% to 91%, illustrating a growth of 10.98%, showing improved and efficient flow of air in the fuel. The results also revealed significant characteristics of combustion, with the cylinder pressure at maximum load, recording an increase from 70.2 bar to 77.3 bar, marking a progress of 10.11% and heat release rate, exhibiting a rise of 18.26% (increased to 48.63 kJ/m^3^deg from 41.11 kJ/m^3^deg). In addition to the above features staying high on performance, the CO emissions was found to be plumped by 69.46% (0.334% to 0.102%), followed by the Hydrocarbons declined by 43.24% (148ppm to 84ppm), CO_2_ dropped by 7.77% (10.3% to 9.5%) and smoke opacity dipped by 14.18% (69.1% to 59.3). It was evidently observed that the hydrogen blend algae biodiesel enhanced a better combustion process by the removal of incomplete combustion areas and enhancing the overall performance and efficiency of the engine. It is noted that while the outcomes of the emissions reveal substantial enhancements, NO_X_ emissions do increase by 47.64% (from 1503ppm to 2220ppm). The exhaust gas temperature is decreased marginally from 520 °C to 498 °C, marking a reduction of 4.23%. The study recommends exploring the integration of hydrogen with other approaches and methods such as EGR, water injection, addition of nano additives and retarded injection timing that would effectively and drastically mitigate NOx emissions. On the whole, the incorporation of A30 and hydrogen would emerge as the most eco-friendlier and the best substitute to pure diesel.

## Data Availability

Data sets generated during the current study are available from the corresponding author M. Selvam (selvamsmkm25@gmail.com) on reasonable request.

## Abbreviations

D100: Pure Diesel Fuel
A30: 30% Botryococcus braunii biodiesel + 70% diesel blend
A100: 100% Botryococcus braunii biodiesel
BTE: Brake Thermal Efficiency
SFC: Specific Fuel Consumption
CA: Crank Angle
A30 + H_2_: 30% Botryococcus braunii biodiesel + 70% diesel with hydrogen gas blends
KOH: Potassium hydroxide
CO: Carbon Monoxide emission
CO₂: Carbon Dioxide emission
CRDI: Common Rail Direct Injection
CV: Calorific Value
EGT: Exhaust Gas Temperature
FAMES: Fatty Acid Methyl Esters
FFAS: Free Fatty Acids
FTIR: Fourier Transform Infrared Spectroscopy
HC: Hydrocarbons emission
NHRR: Net Heat Release Rate
H₂: Hydrogen
LPM: Litres Per Minute (Hydrogen Flow Rate)
NOx: Nitrogen Oxides emission
O₂: Oxygen
Ppm: Parts Per Million
PWM: Pulse Width Modulation
SR: Stoichiometric Ratio
TDC: Top Dead Center
VE: Volumetric Efficiency
DI: Direct Injection
ECU: Electronic Control Unit
SS-316: Stainless Steel Grade 316 (hydrogen-compatible piping material)
RSM: Response Surface Methodology
kg/kWh: Kilogram per kilowatt-hour
kJ/m³ deg: Kilojoule per cubic meter per degree
kJ/kg: Kilojoule per kilogram
kg/m³: Kilogram per cubic meter
mm²/s: Square millimeter per second
